# Case report: A successful second autologous hematopoietic stem cell transplantation in refractory systemic sclerosis, with positive effect on skin involvement, pulmonary function and microcirculation

**DOI:** 10.3389/fimmu.2022.925776

**Published:** 2022-11-23

**Authors:** D.A. Haverkort, B.E. Kersten, A. van Rhenen, W.J.F.M. van der Velden, M.C. Vonk

**Affiliations:** ^1^ Department of Rheumatology, Radboud University Medical Center, Nijmegen, Netherlands; ^2^ Department of Hematology, University Medical Center, Utrecht, Netherlands; ^3^ Department of Hematology, Radboud University Medical Center, Nijmegen, Netherlands

**Keywords:** systemic sclerosis, stem cell transplant (SCT), nailfod capillaroscopy, Rodnan skin score, interstitial lung disease, microvascular abnormalities

## Abstract

**Background:**

Systemic sclerosis (SSc) is a complex autoimmune disease characterized by inflammation, vasculopathy and fibrosis of the skin and internal organs. Treatment with autologous hematopoietic cell transplantation (HCT) for progressive SSc has improved overall and event-free survival rates significantly, but unfortunately disease progression after HCT is seen in a subset of patients. Data on the efficacy and safety of second HCT is scarce.

**Case:**

We present a patient with diffuse cutaneous SSc and associated interstitial lung disease (ILD) who successfully underwent a second HCT for progressive disease five years after a first HCT. We describe changes in skin involvement and pulmonary involvement as well as the changes observed in sequential nailfold microcapillaroscopy (NCM), performed from first presentation up to this moment.

**Conclusion:**

This case adds to the current limited literature on efficacy and safety of a second HCT in SSc refractory cases. Furthermore it outlines the potential of HCT on amelioration of microvasculopathy in SSc.

## Introduction

Systemic sclerosis (SSc) is a complex autoimmune disease characterized by inflammation, vasculopathy and fibrosis of the skin and internal organs. Scleroderma-related interstitial lung disease (SSc-ILD), pulmonary hypertension and cardiac involvement are the leading causes of death among patients with severe SSc (1). To date, no curative treatment is available, but autologous hematopoietic stem cell transplantation (HCT) as treatment for progressive SSc has improved overall and event-free survival rates significantly (2–4). HCT is considered standard-of-care in selected subpopulations of patients with SSc (5). HCT aims to ablate the adaptive immune system and restore normal immune regulation. Unfortunately, disease progression after HCT is seen in a subset of patients, with numbers ranging from 12% after two years up to 40% after 10 years (6, 7). In these cases treatment options are often limited and treatment with immunosuppression including mycophenolate mofetil and cyclophosphamide is usually reintroduced. Data on a second HCT in refractory cases is scarce and only two case report have been published to date (8, 9).

## Case description

A 42-year-old man presented with a 3 month history of Raynaud’s phenomenon (RP), skin thickening and pain in multiple joints. He had a unremarkable past medical history and did not use any medication. He was a cigarettes smoker at the time and had a 30 pack-year smoking history.

On presentation he had diffuse skin thickening with a modified Rodnan skin score (mRSS) of 13, multiple fingertip pitting scars and oligoarthritis of both wrists and elbows. Laboratory tests revealed a slightly elevated erythrocyte sedimentation rate (ESR) of 28 (reference <15 mm/h), positive antinuclear antibodies (ANA) and anti-topoisomerase I antibodies. Nailfold capillary microscopy (NCM) showed a late scleroderma pattern with decreased capillary density and abnormal capillaries. A high-resolution CT (HRCT) of the chest and echocardiogram showed no cardiopulmonary involvement. The diagnosis of diffuse cutaneous systemic sclerosis (dcSSc) was established and he was counselled to quit smoking.

**Figure 1:**
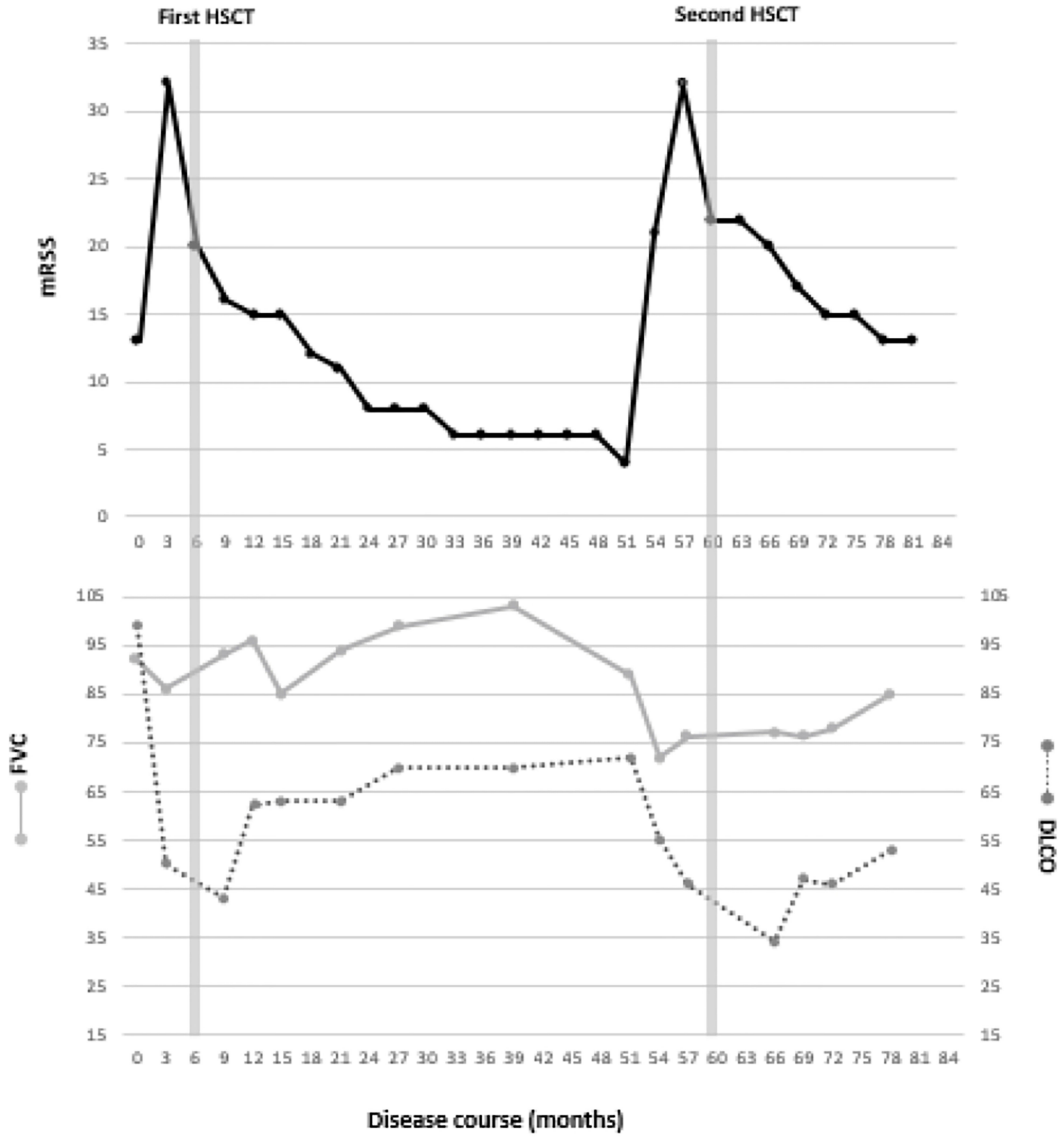
mRSS and pulmonary function.

Treatment was initiated with intravenous cyclophosphamide (750mg/m^2^) every 4 weeks. RP was treated with nifedipine retard 20mg once-daily and for heartburn complaints omeprazole 20mg once-daily was initiated. His arthritis was treated with prednisolone 10mg once-daily. However, after 3 cycles of cyclophosphamide his disease had progressed with worsening of the mRSS to 32 and development of limited SSc-ILD, diagnosed by pulmonary function tests and HRCT-scan (10). The pulmonary function test results and mRSS values are shown in **Figure 1**.

Given his poor prognosis, after counseling the patient and his partner, it was jointly decided to intensify treatment and perform a HCT. Before stem cell mobilization, he was referred to a fertility clinic to perform semen cryopreservation because of the increased risk of infertility following high-dose alkylating agent conditioning. The procedure of the HCT was performed as described before, including the same medication dosing regimen (3). In brief, peripheral blood hematopoietic stem cells were mobilized with intravenous cyclophosphamide 2000mg/m^2^ and G-CSF (filgrastim). After conditioning with cyclophosphamide 200mg/kg, intravenous rabbit antithymocyte globulin (rATG, Genzyme) 7.5 mg/kg and intravenous methylprednisolone 1 mg/kg, CD34+, stem cells were reinfused. The procedure was complicated by neutropenic fever based on a central line-associated bloodstream infection, for which he received broad-spectrum antibiotic treatment.

After the HCT patient recovered very quickly. His skin involvement as measured by mRSS decreased and his pulmonary function test results improved over time (**Figure 1**). Patient remained in a drug-free remission for 4 years. Unfortunately he failed to quit smoking.

Four years after his HCT patient complained of fatigue and worsening of his RP with stable mRSS. But 4 years and 3 months post-HCT the patient returned to our outpatient clinic and showed clinical signs of a relapse of his SSc with severe RP, tendon friction rubs and deterioration of pulmonary function test results and mRSS. A new HRCT of the chest showed increase of ground glass opacities, consistent with progression of SSc-ILD. Treatment with mycophenolate mofetil 1500mg twice-daily was initiated, but was evaluated as ineffective after 3 months as the mRSS increased and pulmonary function tests showed decreased values. Treatment with i.v. Rituximab was considered (11), as well as a second HCT.

Since there is almost no evidence for a second HCT in SSc in terms of safety and efficacy, this treatment is experimental, which we discussed with the patient. We also counselled him about the possible risks of a second HCT including the increased risk of treatment-related mortality. This time, patient managed to quit smoking, which was a strict requirement.

Nearly 5 years after the first HCT, a second HCT was performed. The same protocol for stem cell mobilization and conditioning was used, except for the use of a different type of rATG (Fresenius) because of the risk of sensibilization. There were no adverse events during the procedure. Treatment with cyclosporine was initiated directly post-HCT. *Pneumocystis jirovecii pneumonia* (PJP) prophylaxis with co-trimoxazole was given up to 1 year post-HCT. Because serology showed cytomegalovirus (CMV) IgG positivity, post-HCT treatment with valganciclovir 900mg once-daily was given for prevention of CMV reactivation.

Within 3 months after the second HCT, again significant improvement of mRSS and pulmonary function was seen (**Figure 1**). 6 months post-HCT, cyclosporine was discontinued and treatment with mycophenolate mofetil was initiated. The HRCT scan 1 year after the second HCT showed an decrease in the amount of ground glass opacities and fibrotic changes. At this moment the second HCT was performed 2 years ago and the disease is still in remission with mycophenolate mofetil in a dose of 2 times 1000 mg daily.

## Changes in nailfold capillary microscopy

Vasculopathy is one of the key features of SSc and occurs early in the disease course. NCM allows visualization of microvascular damage and is a useful tool for follow up of SSc microangiopathy. Typical findings on NCM in patients with SSc include loss of capillaries, hemorrhages, giant capillaries, neovascularization and avascular areas (12). Based on these findings, NCM scleroderma patterns can be classified as “early”, “active” or “late” (13). An early pattern is defined by relatively well-preserved capillary distribution, presence of giant capillaries, few capillary hemorrhages and no evident loss of capillaries. The active pattern is specified by frequent giant capillaries and capillary hemorrhages, moderate capillary loss and mild capillary architecture disorganization. A late pattern is characterized by irregular enlargement of capillaries, few or absent giant capillaries, severe capillary loss with avascular areas, ramified/bushy capillaries and disorganization of the normal capillary array (13).

In our patient, NCM images were obtained at baseline, 6 and 9 months after the first HCT, before the second HCT and 6, 9, 12 and 18 months after the second HCT. NCM images were acquired using an optical probe video capillaroscope equipped with a 200x contact lens and connected to image analysis software (at time of the first HCT VideoCap 3.0, at the time of the second HCT Optilia OP-120 011, Mediscope Digital, videomicroscope (USB interface), OptiPix Capillaroscopy software, clinic 1.7.x with a 200x high resolution objective lens with unpolarized light). Two images per finger were evaluated and consisted of both quantitative and qualitative parameters, as described by the by the EULAR Study Group on Microcirculation in Rheumatic Diseases/Scleroderma Clinical Trials Consortium on Capillaroscopy (14). Evaluation of images was performed by 2 experienced physicians (MV and BK). Consensus was reached on every visit. Mean capillary density was calculated by taking the mean capillary density of 2 images of every finger and adding all means, divided through the number of evaluable fingers. The pattern of the nailfold capillaroscopy was determined as described earlier (13).

**Figure 2:**
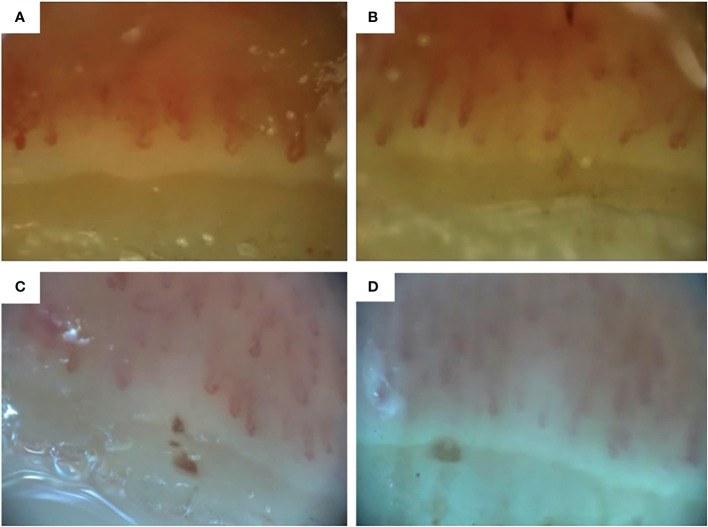
Nailfold capillaroscopic images of digit 4 right **(A)** before first hematopoietic stem cell transplantation (HCT), **(B)** 6 months after the first HCT, **(C)** before the second HCT and **(D)** 6 months after the second HCT. Images A and B obtained with Videocap 3.0, images C and D with Optilia.

NCM findings throughout the disease course are shown in **Table 1** and NCM images before and after the first and second HCT are shown in **Figure 2**. Normal capillary density is defined as >9 capillaries/mm^2^ (13). At baseline, the NCM images showed a late pattern, with neoangiogenesis and severe capillary loss. Mean capillary density at baseline was 3.2 capillaries/mm^2^. 6 and 9 months after the first HCT, the NCM images showed an active pattern with the presence of giant capillaries and a mean capillary density of 6 capillaries/mm^2^ and 7.5 capillaries/mm^2^ respectively. Shortly before the second HCT, the NCM images showed a late scleroderma pattern, with severe capillary loss and a capillary density of 3.8 capillaries/mm^2^. 6 months after the second HCT, NCM images again showed an active pattern with a capillary density of 5 capillaries/mm^2^. 9, 12 and 18 months after the second HCT, NCM images showed a late scleroderma pattern with gradual deterioration of capillary density.

**Table 1:** Capillary density and NCM pattern at baseline and after 1^th^ and 2^nd^ HCT.

	Baseline	1^th^ HSCT			2^nd^ HSCT			
		After 6 months	After 9 months	Before 2^nd^ HSCT	After 6 months	After 9 months	After 12 months	After 18 months
**Mean capillary density (capillaries/mm^2^)**	3.2	6	7.5	3.8	5	4.8	3	3
**NCM Pattern**	Late	Active	Active	Late	Active	Late	Late	Late
**Worst capillary density measured (capillaries/mm^2^)**	2	4	6	2	4	2	3	3

## Discussion

HCT has improved event-free survival and all-cause mortality in SSc, despite the treatment related mortality up to 10% (2–7). HCT aims to eradicate autoreactive T- and B-cells by high dose immunosuppression and promote reconstitution of the immune system by infusion of autologous hematopoietic stem cells. However, it remains unclear whether HCT results in temporary immunosuppression or restores normal immune regulation (15). For patients with disease progression after HCT, treatment options are limited. Conservative treatment with immunosuppressants including mycophenolate mofetil and cyclophosphamide is usually reintroduced. In addition, targeted B-cell therapy with rituximab might be considered. Rituximab represents a promising strategy for skin and lung fibrosis in SSc but long term data is missing and in fact, to date, this is an experimental treatment also (11, 14, 16). Furthermore, treatment with Nintedanib was discussed but declined as this has not shown effect on skin involvement. Tocilizumab treatment was considered inappropriate as the positive effects of tocilizumab is only shown in patients with short disease duration (17). Data on the safety and efficacy of a second HCT in refractory SSc cases is scarce and to our knowledge this is the third case report to date.

The aim of HCT in hematological malignancies is different from SSc. In hematological malignancies, HCT is employed for consolidation of disease remission after induction with chemotherapy. In multiple myeloma, indication for a second HCT is limited to patients with upfront tandem autologous transplantation and to patients that have a late relapse in second remission and with a good performance status and limited comorbidities (18, 19). In general, a second HCT comes with more complications and increased risk of secondary malignancies and cardiotoxicity due to more exposure to alkylating agents. Also difficulties with stem cell mobilization can occur and development of hypersensitivity reactions to ATG can result in serum sickness and anaphylaxis.

In contrast to allogeneic HCT, there is no role for maintenance immunosuppressive therapy after autologous HCT as there is no risk of graft versus host disease. In SSc, event-free survival without immunosuppressive therapy is seen in approximately 80% of patients at 5 (3, 6) and 66% at 15 years after HCT (6). However, after a second HCT long-term T-cell suppression is sought, to prevent another relapse of the autoimmune disease. Similar to the knowledge from patients treated with an allogeneic HCT maintenance of T-cell suppression in our patient was initiated after the second HCT with cyclosporine and mycophenolate mofetil treatment (20). The risk of long-term effects of continuous immunosuppressive therapy, such as the increased risk of malignancies and organ damage, must be taken into account but don’t seem to outweigh the expected benefits.

Long term efficacy of HCT in improving skin thickness, forced vital capacity (FVC) and health-related quality of life in SSc has been demonstrated consistently (2–7). This case shows the potential benefit of a second HCT with post HCT immune suppression after relapse after a previous HCT. Less is known of the effect of HCT on microvasculature. Vasculopathy is one of the key features of SSc. Reversible vasospasm of digital arteries causes the appearance of Raynaud’s phenomenon. Hypoxia and reduction of endothelial progenitor cells cause microvascular damage leading to the appearance of digital ulcers and pitting scars. NCM patterns reflect the evolution of the disease process and capillary density is considered the most strong predictor for identifying SSc patients who will develop severe disease (13). There is no association between smoking and smoking cessation and NCM patterns (21). Several studies have shown that NCM abnormalities and particularly capillary loss in SSc patients change over time and correlate with clinical disease severity (22, 23). Also, the influence of immunomodulating drugs on the improvement of microcirculation measured by NCM has been described (11). Changes in NCM patterns after HCT have been observed in 7 patients with SSc previously, with improvement seen in all patients within 3 months and up to 2 years post-HCT (24, 25).

In our patient we observed changes in NCM patterns, with regression from late to active SSc pattern and recovery of capillary density. This indicates that HCT influences the microvasculature and promotes vascular remodeling, which is not seen in patients with conventional treatment (24).

To date, several international collaborations aim to evaluate the value of NCM as a biomarker for disease progression or development of disease complications in SSc (26, 27). Especially a reduced capillary density is associated to the development of both interstitial lung disease and pulmonary hypertension, although not all studies confirm this association (25). The capillary density of our patient increased after the first HCT, but decreased again to very low values before the second HCT. Our patient suffered from increased RP at the time of relapse of his disease. **Figure 1** shows his improvement of skin involvement after both the first and the second HCT but a slower and partial improvement of pulmonary function. Although our patient had an improvement of his capillary density after the second HCT it was not as strong as after the first HCT. An explanation for this reduced effect on capillary density after the second HCT compared to after the first HCT could be the much longer disease duration, the smaller improvement of the pulmonary function or the failure of normalization of endothelial precursor cells after the second HCT (24, 28).

In conclusion, this case highlights the option of a second HCT can be considered in relapsed SSc. Improvement of skin involvement and pulmonary function can be achieved, as well as amelioration of microvasculopathy. What the clinical value of improvement of the microvasculature is, has to be determined to date. However, the changes seen in NCM patterns with recovery of capillary density, emphasizes that NCM might be a promising biomarker in SSc.

## Patients perspective

The patient and his partner were fully engaged throughout the treatment process. The potential risks and benefits of a second HCT were discussed extensively. The patient agreed with publication of this case report and a written informed consent was obtained.

## Data availability statement

The raw data supporting the conclusions of this article will be made available by the authors, without undue reservation.

## Ethics statement

Ethical review and approval was not required for the study on human participants in accordance with the local legislation and institutional requirements. The patients/participants provided their written informed consent to participate in this study.

## Author contributions

MV was the treating physician throughout the entire disease course. AR and WV were involved in the HCT treatment. MV and BK performed evaluation of the NCM images. DH wrote the first draft of the manuscript. MV wrote sections of the manuscript. All authors contributed to the article and approved the submitted version.

## Conflict of interest

The authors declare that the research was conducted in the absence of any commercial or financial relationships that could be construed as a potential conflict of interest.

## Publisher’s note

All claims expressed in this article are solely those of the authors and do not necessarily represent those of their affiliated organizations, or those of the publisher, the editors and the reviewers. Any product that may be evaluated in this article, or claim that may be made by its manufacturer, is not guaranteed or endorsed by the publisher.
